# Control of the gut microbiome by fecal microRNA

**DOI:** 10.15698/mic2016.04.492

**Published:** 2016-03-09

**Authors:** Shirong Liu, Howard L. Weiner

**Affiliations:** 1 Department of Neurology, Ann Romney Center for Neurologic Diseases, Brigham and Women’s Hospital, Harvard Medical School, Boston, MA 02115, USA.

**Keywords:** microbiota, microRNA, host-microbe interaction, dysbiosis, colitis

## Abstract

Since their discovery in the early 90s, microRNAs (miRNAs), small non-coding RNAs, have mainly been associated with posttranscriptional regulation of gene expression on a cell-autonomous level. Recent evidence has extended this role by adding inter-species communication to the manifold functional range. In our latest study [Liu S, *et al.*, 2016, Cell Host & Microbe], we identified miRNAs in gut lumen and feces of both mice and humans. We found that intestinal epithelial cells (IEC) and Hopx+ cells were the two main sources of fecal miRNA. Deficiency of IEC-miRNA resulted in gut dysbiosis and WT fecal miRNA transplantation restored the gut microbiota. We investigated potential mechanisms for this effect and found that miRNAs were able to regulate the gut microbiome. By culturing bacteria with miRNAs, we found that host miRNAs were able to enter bacteria, specifically regulate bacterial gene transcripts and affect bacterial growth. Oral administration of synthetic miRNA mimics affected specific bacteria in the gut. Our findings describe a previously unknown pathway by which the gut microbiome is regulated by the host and raises the possibility that miRNAs may be used therapeutically to manipulate the microbiome for the treatment of disease.

## Normal gut microbiota is essential for health

It is known that the mammal gut harbors trillions of commensal microbes, which are host specific and are important for health. Disturbances of the microbiome have been reported to have consequences for immune development, metabolism, and a variety of diseases states including autoimmune diseases, autism and cancer. Thus, it is important to understand how the microbiome is regulated and to identify strategies to manipulate it.

## Fecal miRNAs

Studies have shown that miRNAs can exist extracellularly in the body and might be indicative of specific diseases. Hence, our primary research focus are extracellular miRNAs in the circulation as biomarkers of multiple sclerosis. We then asked whether extracellular miRNAs were present in the feces as well, and found that miRNAs were part of the normal composition of feces both in mice and humans. We compared the relative abundance of miRNAs in the upper level and lower level of the gut, in germ-free mice and in antibiotic treated mice. Intriguingly, we found that the abundance of miRNA was inversely correlated with the abundance of microbes. This suggested that microbes might take up miRNAs and that these miRNAs might in turn affect microbes.

## miRNA affects bacterial gene expression and bacterial growth

We cultured bacteria with fluorescence labeled miRNA mimics and observed that the miRNAs entered bacteria. Specifically, they co-localized with bacterial nucleic acids, which provide the temporal and spatial basis for bacterial gene expression regulation. We then aligned the bacterial gene sequences to the miRNA database. Unexpectedly, we found that all bacterial gene sequences we queried were found to pair with different miRNAs, and these miRNAs were derived from host species including *C. elegans*, *Drosophila*, and mammals (mice and humans). To ask whether miRNAs altered bacterial gene expression, we synthesized miRNA mimics and added them to the culture of bacteria such as *F. nucleatum* and *E. coli*. We found that the transcripts of the bacterial genes were specifically altered upon miRNA treatment and that bacterial growth was affected by miRNA treatment.

## miRNA shapes gut microbiota

We then asked whether this novel mechanism of regulation could shape the composition of gut microbiome in the host. To address this, we first identified the primary sources of fecal miRNAs to be intestinal epithelial cells (IECs) and Hopx-expressing cells by generating mice, with a cell-type-specific deficiency in the miRNA processing enzyme, Dicer. Data from 16S rDNA sequencing suggested that IEC-specific miRNA knockout (*Dicer1*^∆^*^IEC^*) caused dysbiosis of the gut microbiota. This was accompanied by the worsening of colitis in a dextran sulfate sodium (DSS) model. When we isolated the fecal miRNA from wild-type mice and transferred them to *Dicer1*^∆^*^IEC^* recipient mice by gavage, the gut microbiota in the recipient mice was restored to the wild-type phenotype and the colitis was ameliorated. Finally, we synthesized the miRNA mimic for miR-1226 and found that it affected growth of *E.coli* *in vitro* and when given orally to mice also affected *E.coli in vivo.*

## Conclusions and discussion

**Figure 1:**
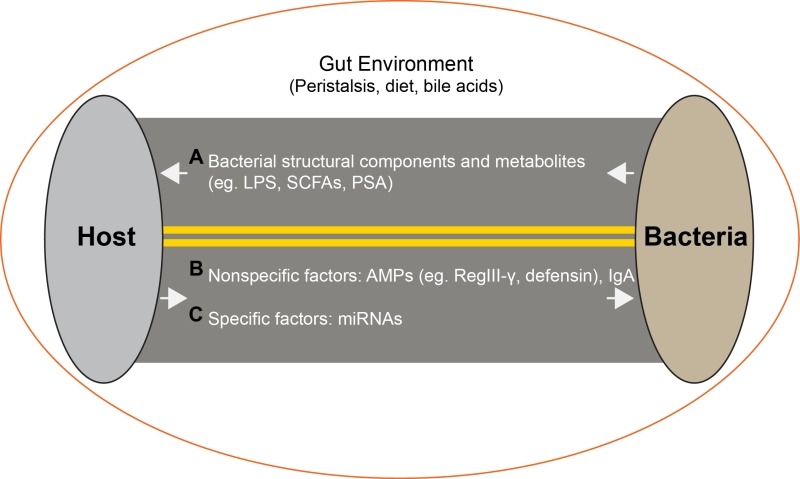
FIGURE 1: Microbe-host interactions in the gut lumen. **(A)** Bacterial to host mediators. **(B)** Non-specific host to bacterial mediators. **(C)** Specific host to bacteria mediators.

The gut microbiome plays an important role in health and disease. Commensals reside in an external environment (i.e., the intestinal lumen), and they interact with the environment and host through mechanisms that are fundamentally different from systemic compartments. The host-microbe interaction is a two-way interaction (Figure 1) conditioned by diet, bowel peristalsis and digestive enzymes. Studies to date have focused on how microbes affect the host through factors such as microbial components, metabolites such as LPS, short chain fatty acids (SCFAs) and polysaccharide A (PSA) (Figure 1, A). The effect of the host on microbes includes antimicrobial peptides (AMPs) such as RegIII-γ, defensin. These mechanisms are largely non-specific (Figure 1, B). Secreted IgA is involved in maintaining the microbiota, but its importance is not clear since IgA immunodeficiency had little effect on the composition of the gut flora. Here, we have identified host miRNAs that are able to specifically modulate the gut microbiome (Figure 1, C). Our findings identify a novel mechanism by which one could specifically manipulate the gut microbiome for the treatment of disease.

